# Developing a pragmatic consensus procedure supporting the ICH S1B(R1) weight of evidence carcinogenicity assessment

**DOI:** 10.3389/ftox.2024.1370045

**Published:** 2024-04-05

**Authors:** Arianna Bassan, Ronald Steigerwalt, Douglas Keller, Lisa Beilke, Paul M. Bradley, Frank Bringezu, William J. Brock, Leigh Ann Burns-Naas, Jon Chambers, Kevin Cross, Michael Dorato, Rosalie Elespuru, Douglas Fuhrer, Frances Hall, Jim Hartke, Gloria D. Jahnke, Felix M. Kluxen, Eric McDuffie, Friedemann Schmidt, Jean-Pierre Valentin, David Woolley, Doris Zane, Glenn J. Myatt

**Affiliations:** ^1^ Innovatune srl, Padova, Italy; ^2^ Takeda Development Center Americas, San Diego, CA, United States; ^3^ Independent Consultant, Kennett Square, PA, United States; ^4^ Toxicology Solutions, Inc., Marana, AZ, United States; ^5^ Instem, Cambridge, United Kingdom; ^6^ Chemical and Preclinical Safety, Merck Healthcare KGaA, Darmstadt, Germany; ^7^ Brock Scientific Consulting, LLC, Hilton Head, SC, United States; ^8^ Magnolia Toxicology Consulting, LLC, Traverse City, MI, United States; ^9^ Instem, Conshohocken, PA, United States; ^10^ MBX Biosciences, Carmel, IN, United States; ^11^ Independent Consultant, Annapolis, MD, United States; ^12^ BioXcel Therapeutics, Inc., New Haven, CT, United States; ^13^ Gilead Sciences, Inc., Foster City, CA, United States; ^14^ Independent Consultant, Chapel Hill, NC, United States; ^15^ ADAMA Deutschland GmbH, Cologne, Germany; ^16^ Neurocrine Bioscience, Inc., San Diego, CA, United States; ^17^ Sanofi-Aventis Deutschland GmbH, Frankfurt, Germany; ^18^ UCB Biopharma SRL, Braine l’Alleud, Belgium; ^19^ ForthTox Ltd., Linlithgow, United Kingdom

**Keywords:** carcinogenicity assessment, WoE, ICHS1B, 2-year rat bioassay, integrated assessment, pharmaceuticals, drug development

## Abstract

The ICH S1B carcinogenicity global testing guideline has been recently revised with a novel addendum that describes a comprehensive integrated Weight of Evidence (WoE) approach to determine the need for a 2-year rat carcinogenicity study. In the present work, experts from different organizations have joined efforts to standardize as much as possible a procedural framework for the integration of evidence associated with the different ICH S1B(R1) WoE criteria. The framework uses a pragmatic consensus procedure for carcinogenicity hazard assessment to facilitate transparent, consistent, and documented decision-making and it discusses best-practices both for the organization of studies and presentation of data in a format suitable for regulatory review. First, it is acknowledged that the six WoE factors described in the addendum form an integrated network of evidence within a holistic assessment framework that is used synergistically to analyze and explain safety signals. Second, the proposed standardized procedure builds upon different considerations related to the primary sources of evidence, mechanistic analysis, alternative methodologies and novel investigative approaches, metabolites, and reliability of the data and other acquired information. Each of the six WoE factors is described highlighting how they can contribute evidence for the overall WoE assessment. A suggested reporting format to summarize the cross-integration of evidence from the different WoE factors is also presented. This work also notes that even if a 2-year rat study is ultimately required, creating a WoE assessment is valuable in understanding the specific factors and levels of human carcinogenic risk better than have been identified previously with the 2-year rat bioassay alone.

## 1 Introduction

**FIGURE 1:**
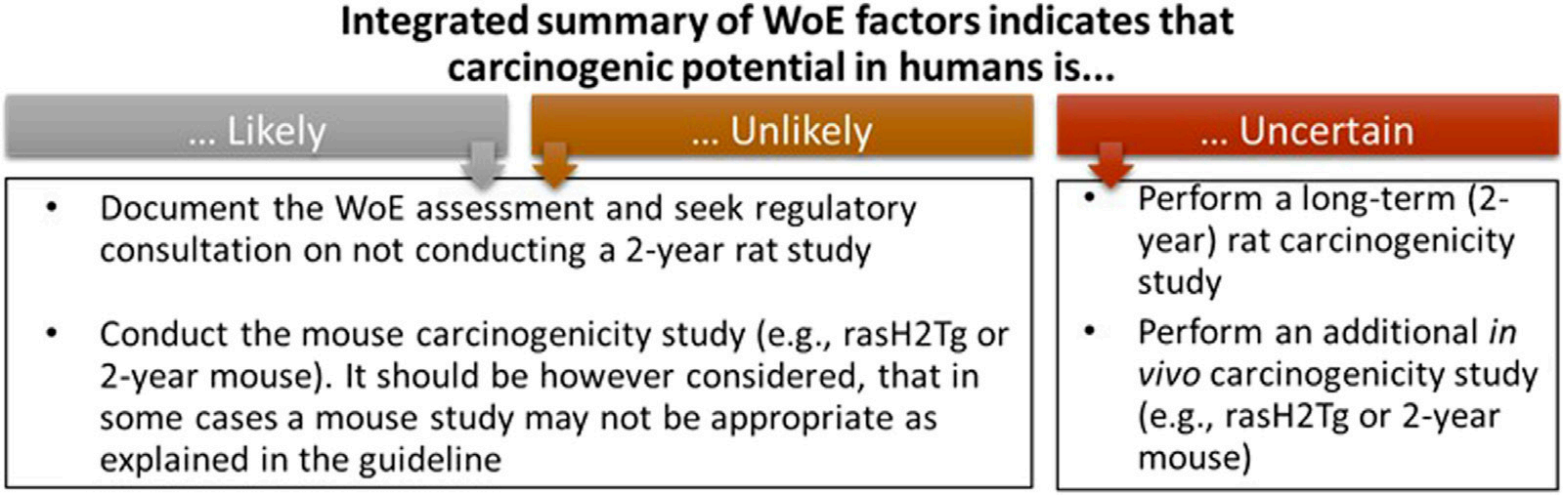
The three outcomes of the WoE integrated assessment as defined by the ICH S1B(R1) guideline. These outcomes and actions provide a basis for Sponsors to define project goals for logistics around how to best fit the WoE approach into the project timeline so that the decision that a 2-year rat study is needed does not result in a major impact to the project timeline.

The International Council on Harmonization (ICH) S1B(R1) guideline provides a framework for evaluating the carcinogenic potential of pharmaceuticals to enhance the assessment of human carcinogenic risk, increasing efficiency and consistency in testing approaches across regulatory agencies. The original guideline was revised in 2022 and adopted across multiple regulatory jurisdictions (ICH S1B(R1), 2022). The addendum of this guideline introduces a detailed weight of evidence (WoE) approach supporting a robust scientific strategy for assessing human carcinogenic risk of pharmaceuticals. The addendum identifies six WoE factors to assess whether conducting a 2-year rat carcinogenicity study (bioassay) would add value to the existing data supporting a human carcinogenicity risk assessment. In certain cases (Figure 1), the fully integrated WoE approach is proposed as a potential alternative to the 2-year rat bioassay thus reducing animal testing without compromising human safety. This pivotal change introduced in the ICH S1B(R1) guideline is expected to increasingly rely on new and alternative strategies for determining carcinogenic risk. This is in line with the 3Rs [Replacement, Reduction, and Refinement (Russell and Burch, 1959)] approach of animal use in science (Van Der Laan et al., 2023), that is embraced by several programs. For example, the FDA Modernization Act 2.0 gives the drug development industry the option to use alternatives to animal testing to determine safety and efficacy of drugs, empowering the use of innovative non-animal methods in the most rigorous and scientific way (US Congress, 2022; Wadman, 2023). Furthermore, there are calls from members of the European Parliament to accelerate the transition to an animal-free research and testing (EU, 2021), which is also being mapped by the European Food Safety Authority (EFSA) (Escher et al., 2022; Cattaneo et al., 2023), the European Chemicals Agency (ECHA) (ECHA, 2023) and the European Medicines Agency (EMA) (EMA, 2020).

Rodent carcinogenicity studies of pharmaceuticals are usually initiated in the late drug development phase, following the completion of shorter repeat-dose toxicity studies (which are used as dose ranging studies for the 2-year rat bioassay) and Phase I and Phase II clinical trials. The rat carcinogenicity study is usually the last nonclinical study completed prior to submission of the Marketing Application. Without intent to extend the drug development timeline, the novel strategy described in the ICH S1B(R1) guideline encourages early planning of carcinogenicity assessment based on the integration and combination of relevant evidence from standard *in vitro* and *in vivo* studies. It also highlights the use of additional investigative approaches to address concerns and data gaps identified by the WoE evaluation. The outcome of the WoE assessment is a determination whether a 2-year rat study adds value after all the data (including chronic toxicology data) are available, and then agreement with regulators is pursued; therefore it is essential that the approach to the WoE assessment is planned timely so that decision regarding the need for a 2-year rat study can be achieved early enough; consequently, if needed, the bioassay can be started without major impact to the project timeline.

The integrated WoE approach that applies to molecules requiring carcinogenicity assessment according to ICH S1A (1995) is supported by experience with a similar WoE framework described for biotechnology-derived therapeutics in ICH S6(R1) (2011). The assessment for biotechnological products includes analysis of data from multiple sources, including published data (e.g., information from transgenic, knock-out or animal disease models, and human genetic diseases), information on class effects, detailed information on target biology and mechanism of action, *in vitro* data, chronic toxicity studies, reproductive toxicology studies and clinical data. If this WoE assessment is not sufficient to clearly assess carcinogenicity, under ICH S6(R1), alternative studies can be proposed to reduce remaining uncertainties or to address data gaps and inform more clearly the potential risk.

The ICH S1B(R1) WoE factors should be considered in a holistic and integrative manner to determine the need, timing, and design of carcinogenicity studies in drug development. Accordingly, the factors bring together pharmacological, biological, and toxicological data that can be integrated for human carcinogenicity risk assessment leading to a decision on whether carcinogenic potential of the therapeutic agent in humans is: A) likely and a 2-year rat carcinogenicity study would not add value; B) unlikely and a 2-year rat carcinogenicity study would not add value; or C) uncertain and a 2-year rat carcinogenicity study would add value to the overall safety assessment for humans (Figure 1). The WoE criteria include evidence from public sources and relevant drug development studies, and they cover six different factors described in Table 1: 1) target biology; 2) secondary pharmacology; 3) histopathology from chronic studies; 4) hormonal effects; 5) genotoxicity; and 6) immune modulation. In general, a robust assessment of the absence of concern for all the WoE criteria supports a conclusion that a 2-year rat bioassay would not add value to the overall human carcinogenicity risk assessment. The 2-year rat bioassay is less likely to be of value also in the case of evidence of unequivocal genotoxicity or broad immunosuppression indicating a carcinogenic risk to humans (ICH S1B(R1), 2022). In these cases, the risk can be clearly stated in the product label.

**TABLE 1:** Description of the WoE factors and their interpretation in the WoE assessment as included in the ICH S1B(R1) guideline (ICH S1B(R1), 2022). As discussed in the guideline, decision making is driven by the evidence collected to assess carcinogenic risk from each of the six WoE criteria. The guideline addendum also notes that in addition to cases where all the WoE factors indicate no risk, the 2-year rat bioassay is likely not to add value in the case of unequivocal genotoxicity risk or observed effects of broad immunosuppression.

WoE factor short name	Description^a^	2-year rat study and/or investigative approaches more likely if … ^a^	2-year rat study and/or investigative approaches less likely if … ^a^
Target biology	“Data that inform carcinogenic potential based on drug target biology and the primary pharmacologic mechanism of the parent compound and major human metabolites; this includes drug target distribution in rats and humans along with the pharmacologic activity and potency of the parent compound and major metabolites in these species; available information from genetically engineered models; human genetic association studies; cancer gene databases; and carcinogenicity information on class effects, if available.”	“Poorly characterized biologic pathways, unknown class effects”	“Well characterized biologic pathways, known class effects”
Secondary pharmacology	“Results from secondary pharmacology screens for the parent compound and major metabolites that inform selectivity and off-target potential, especially those that inform carcinogenic risk (e.g., binding to nuclear receptors).”	“Low target selectivity, off-target activity”	“High target selectivity, no off-target activity”
Histopathology chronic studies	“Histopathology data from repeated-dose toxicity studies completed with the compound, with particular emphasis on the 6-month rat study, including plasma exposure margin assessments of parent drug and major metabolites.”	“Hyperplastic or other lesions of concern”	“No findings of concern or human-irrelevant findings”
	“Histopathology findings from 6-month rat toxicity studies of particular interest for identifying carcinogenic potential in a 2-year rat study include cellular hypertrophy, cellular hyperplasia, persistent tissue injury and/or chronic inflammation, foci of cellular alteration, preneoplastic changes, and tumors. It is important to provide an understanding of the likely pathogenesis, and/or address the human relevance of such findings. While the 6-month rat toxicity study is the primary study to be used for assessing the likely outcome and value of conducting a 2-year rat study, shorter-term rat studies can sometimes also provide histopathologic conclusions of value. Data from long-term toxicity studies in non-rodents and mice may also be useful for providing additional context on the human relevance of rat study findings (e.g., species-specific mechanistic differences) and whether there is value in conducting a 2-year rat study.”		
Hormonal effects	“Evidence for hormonal perturbation, including knowledge of drug target and compensatory endocrine response mechanisms; weight, gross and microscopic changes in endocrine and reproductive organs from repeated-dose toxicity studies; and relevant results from reproductive toxicology studies, if available.”	“Endocrine/reproductive organ perturbation”	“No findings of concern or human-irrelevant findings”
	“Findings from rat toxicity studies suggesting hormonal perturbation may include microscopic changes in endocrine or reproductive tissues of atrophy, hypertrophy, and hyperplasia and/or biologically significant endocrine and reproductive organ weight changes which are not explained as findings secondary to processes such as stress or altered body weight. Changes of this nature may be considered evidence of functional hormonal perturbation even when changes in hormone levels are not documented. Such findings may be suggestive of potential carcinogenic risk unless investigated for human relevance and demonstrated otherwise.”		
Genotoxicity	“Genetic toxicology study data using criteria from ICH S2(R1) Genotoxicity Testing and Data Interpretation for Pharmaceuticals Intended for Human Use (ICH S2(R1), 2012); equivocal genotoxicity data that cannot be resolved in accordance with ICH S2(R1) recommendations increases uncertainty with respect to the carcinogenic potential.”	“Positive genotoxicity data of uncertain human relevance”	“No genotoxicity risk or unequivocal genotoxicity”
Immune modulation	“Evidence of immune modulation in accordance with ICH S8 Immunotoxicity Studies for Human Pharmaceuticals (ICH S8, 2006). Evidence of broad immunosuppression may provide sufficient concern for human risk that would not be further informed by standard rat and mouse carcinogenicity studies.”	“Immune effects of uncertain human relevance”	“No effects on immune cell/tissues or broad immunosuppression in humans”

^a^ Description and summary interpretation as originally taken from the ICH S1B(R1) guideline.

Notably, the ICH S1B(R1) strategy supports the incorporation of results from different investigative approaches such as molecular biomarkers and emerging technologies and the use of published data on related molecules. Targeted nonstandard clinical data can also be collected in clinical trials to help to address hypothesized concerns of carcinogenic drug actions and determine relevance of animal findings to humans. These additional results can be used to inform the WoE factors and support the decision making on the need and value of conducting the 2-year rat bioassay. The guideline notes that a rasH2-Tg mouse study is not expected to be completed to support a WoE assessment. However, if rasH2-Tg mouse study results are available, they should be included as evidence, and, for example, they can inform the strength of association of target modulation with rodent tumor development when sufficient pharmacologic activity is documented.

The present work leverages the rationale of the *in silico* toxicology protocols initiative (Myatt et al., 2018; Myatt et al., 2022), where an international network of experts has been working to identify principles for generating, recording, communicating, archiving and then evaluating toxicity assessments (employing *in silico* methods when appropriate) in a uniform, consistent and reproducible manner.

The present work proposes a pragmatic standardized procedure framing the ICH S1B(R1) human carcinogenicity assessment in the spirit of the ideas underlying the *in silico* toxicology protocols, thus aiming to make decisions (i.e., on whether a 2-year rat carcinogenicity study adds value) that are transparent, consistent, documented, repeatable and defendable. In general terms, WoE analyses integrate numerous pieces of evidence to make a scientifically defensible conclusion, that may be inherently based on subjective judgment and thus affected by potential bias, as, for example, discussed by the Organisation for Economic Co-operation and Development (OECD) in relation to weight of evidence for chemical assessment (OECD, 2019). Therefore, an established procedure that drives the process of collating, weighing and evaluating such evidence ensures that the analysis and the conclusions are clearly understood, documented and thus transparent to all stakeholders. The pragmatic consensus procedure described here is meant to support the creation of the Carcinogenicity Assessment Document (CAD), which reports the expected utility of the 2-year rat study as derived from the WoE assessment.

Determination in certain infrequent instances of whether a mouse study may not be needed for the carcinogenicity assessment is discussed in ICH S1B(R1) and is not further addressed in this work. Moreover, strategies for exact timing of study activities and regulatory interactions are also considered out of scope of this publication.

## 2 Background

An international network of experts from different organizations has been working to develop *in silico* toxicology protocols for combining evidence coming from different sources (e.g., *in vitro* and *in vivo* experimental data and *in silico* results) and to establish an overall assessment and confidence score for a given toxicological endpoint (Myatt et al., 2018; 2022). In general, a protocol is a standardized procedure that frames the hazard assessment process to facilitate transparent, consistent and documented decision-making. This protocol concept has been applied for genetic toxicology (Hasselgren et al., 2019), skin sensitization (Johnson et al., 2020) and acute oral toxicity (Zwickl et al., 2022), and has been discussed in a number of other publications covering carcinogenicity (Tice et al., 2021), organ toxicity (Bassan et al., 2021a; Bassan et al., 2021b), neurotoxicity (Crofton et al., 2022), and confidence of a general integrated assessment (Johnson et al., 2022). In the present work the *in silico* toxicology protocol concept (Myatt et al., 2018; Myatt et al., 2022) is applied to guide the ICH S1B(R1) assessment.

## 3 Overview of the proposed pragmatic consensus procedure

The *in silico* toxicology protocol approach (Myatt et al., 2018; 2022) is applied here in a more specific fashion to the ICH S1B(R1) WoE assessment, where the endpoint of interest is understanding the added value of a 2-year rat study to the assessment of human carcinogenic risk. There is no “one size fits all” approach for such a novel carcinogenicity assessment strategy and its application must be tailored to the specific pharmaceutical being evaluated and the logistics surrounding the project development timeline. This work attempts to standardize as much as possible the procedure that guides the integration of data associated with the different ICH S1B(R1) WoE criteria (Table 1). The result of this effort is meant to be a pragmatic consensus procedure providing indications and suggestions that guide holistic, science-based and intelligent conclusions as well as facilitating the creation and successful submission of the CAD that would be deemed to be sufficiently comprehensive, objective and balanced, and both reasonable and convincingly conclusive.

The pragmatic consensus procedure is intended to discuss best-practices for both the organization of the studies and presentation of the data in a suitable format as well as to clarify expectations in terms of the types of integrated evidence to be presented in the CAD. Indeed, definition of a reporting format for collected evidence, results and conclusions helps clarify what is expected in terms of the types of evidence to be included and critical questions to be answered.

**FIGURE 2:**
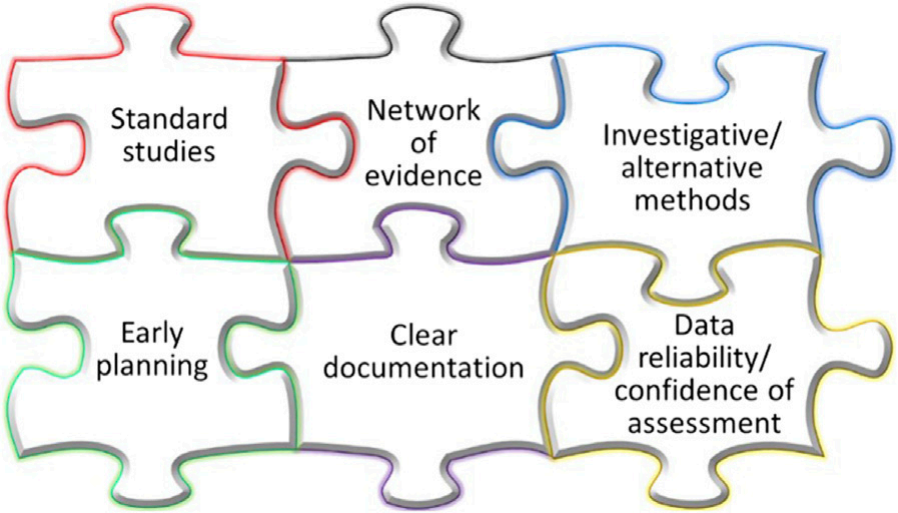
Attributes of the proposed pragmatic consensus procedure for the ICH S1B(R1) integrated assessment.

The procedure contains proposals on: 1) the strategy of the integrative WoE carcinogenicity assessment; 2) approaches for the collection and organization of data and information; 3) analysis of available evidence; 4) reporting of the results. In order to establish a pragmatic consensus procedure for the integrated WoE assessment, several general aspects are considered and examined as summarized in Figure 2 and described below.

### 3.1 Network of evidence

**FIGURE 3:**
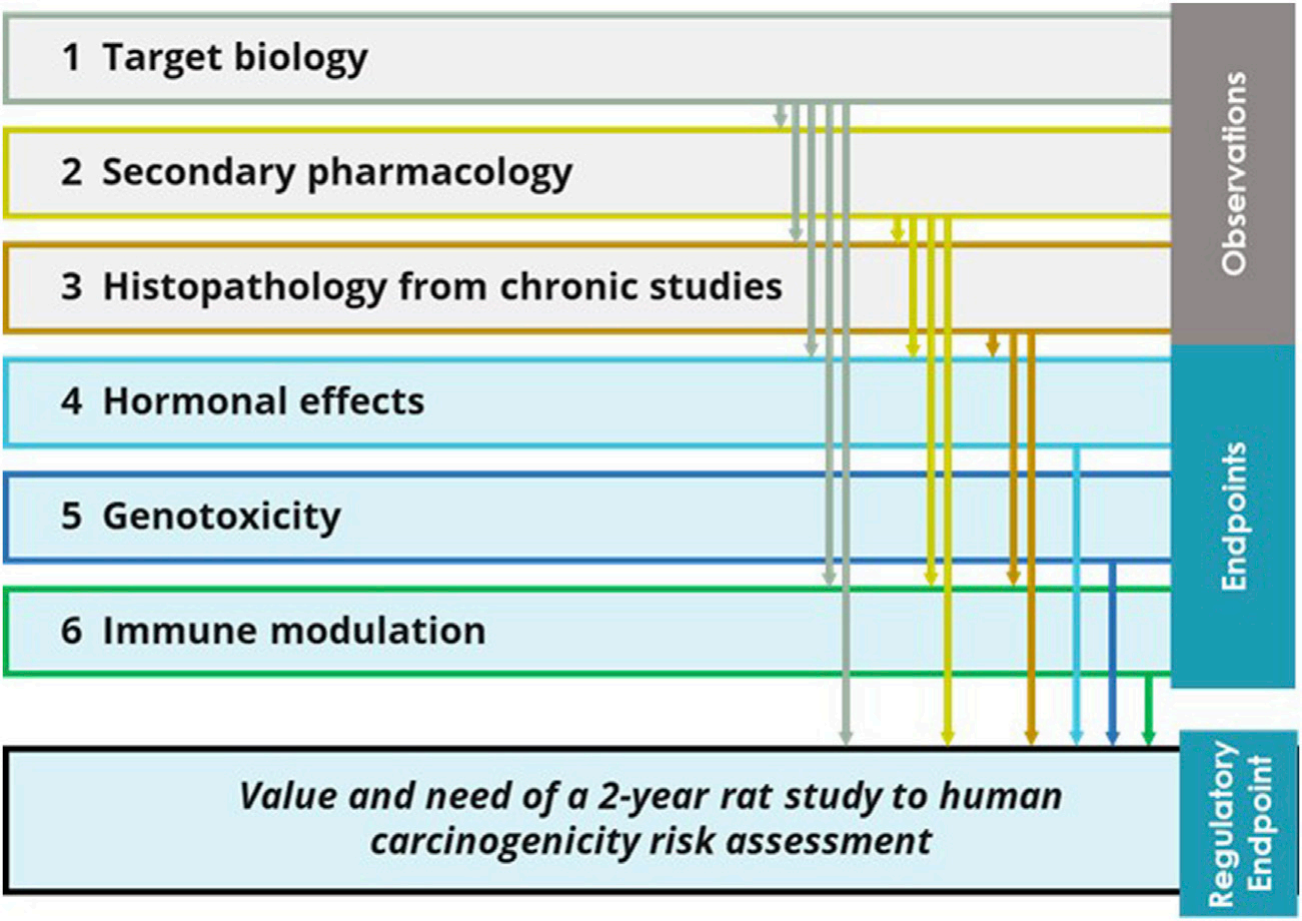
Potential relationship among the six ICH S1B(R1) WoE factors. Observations from the target biology analysis, secondary pharmacology and histopathology from chronic studies provide evidence that can inform the human carcinogenic risk and the added value of the 2-year rat study; such evidence would also inform the other endpoints forming the other WoE factors: hormonal perturbation and immune modulation. In general, the six WoE factors form a network of evidence where the analysis of each WoE factor can be integrated with input from the other WoE factors.

The six ICH S1B(R1) WoE factors are related to each other, since the evidence belonging to a specific WoE area (e.g., histopathology from chronic studies) can be used to inform other WoE criteria (e.g., hormonal effects) as illustrated in Figure 3. Different observations are collected from the analysis of target biology, secondary pharmacology and histopathology from chronic studies. Such observations are integrated with the evaluation of the other endpoints associated with the remaining WoE factors (hormonal effects, genotoxicity, and immune modulation). In general, the assessment of some WoE factors can be supported by evidence and signals collected from other WoE factors. The six WoE factors can thus be viewed as a network of evidence within a holistic assessment framework that is used synergistically to analyze and explain signals (and/or absence of signals), in order to demonstrate that the ICH S1B(R1) integrated assessment has been conducted thoroughly, and that all appropriate aspects of the WoE approach have been considered. For example, a histopathological finding from the 6-month rat study may be connected to data coming from the secondary pharmacology screening to aid interpretation and give a better understanding of the evidence presented based on assessing coherence of observed responses.

### 3.2 Mechanistic analysis

Human relevance of the findings from the different WoE areas needs to be established. Mechanistic analysis of effects of potential concern is critical to determine whether the mode of action is relevant to humans, and to support interpretation of signals and findings (an example will be given further below when discussing chronic inflammation in relation to the histopathology from chronic studies factor in Section 4.3). The Adverse Outcome Pathway (AOP) framework (OECD, 2023) can help to organize the mechanistic understanding that is being built while performing the ICH S1B(R1) integrated assessment. The AOP framework describes a sequence of events that is triggered by an initial interaction between a stressor and a biomolecule (i.e., Molecular Initiating Event, MIE) and can progress through a dependent series of intermediate key events (KEs) involving structural and functional changes. This sequence of events, potentially part of a larger network, ultimately culminates in the adverse outcome (AO) relevant to an organism (OECD, 2017). Existing consensus about a given AOP should be carefully evaluated before using the AOP. Translational mechanistic or safety biomarkers that can reflect animal study findings linked to carcinogenesis and serve as bridges for monitoring for such potential drug actions at therapeutic exposures in clinical trials, are also useful for addressing human relevance.

### 3.3 Alternative methodologies and novel investigative approaches

Evidence sources from *in vivo* studies are primarily from standard toxicology studies on the drug candidate (e.g., histology from subchronic and chronic rodent studies, reproductive toxicology studies and the standard genetic toxicology battery) to the fullest extent possible to minimize the need for additional, unwarranted animal studies. Potential elements of concern identified during the evaluation of the six WoE factors could be further inspected by applying alternative methodologies such as network biology approaches (e.g., Wang, 2022), quantitative systems toxicology (e.g., Bloomingdale et al., 2017), or other novel investigative approaches such as organotypic cultures (e.g., Hayden and Harbell, 2021), organs-on-a-chip (e.g., Ingber, 2022; Leung et al., 2022), humanized mice (e.g., Ye and Chen, 2022), disease models (e.g., Loewa et al., 2023). These approaches are selected as appropriate to improve the mechanistic understanding and to interpret and explain the relevance of findings to humans.

### 3.4 Early planning

**FIGURE 4:**
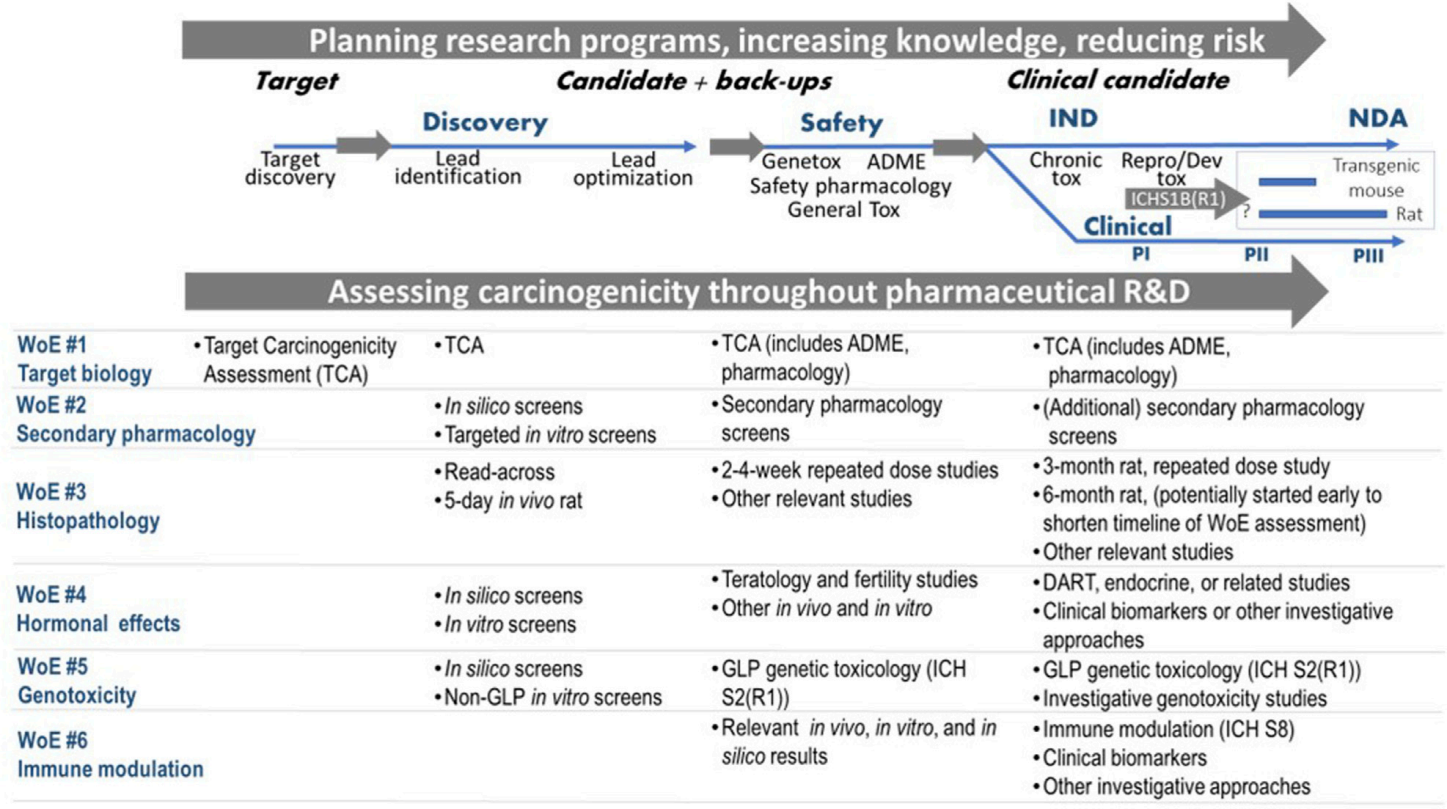
Assessing carcinogenicity throughout pharmaceutical research and development (R&D) incorporating evidence from the ICH S1B(R1) factors. Abbreviations: IND (Investigational New Drug); NDA (New Drug Application); PI (Phase I); PII (Phase II); PIII (Phase III); ADME: absorption, distribution, metabolism and excretion; GLP: Good Laboratory Practice; DART: Developmental & Reproductive Toxicity.

Early, pragmatic and flexible planning of the integrative WoE carcinogenicity assessment is advisable for anticipation of the ICH S1B(R1) assessment as it allows one to capture signals for carcinogenicity concern at an early stage of the drug discovery and development process (i.e., carcinogenic potential is likely) and also to make early decisions as to whether a WoE approach is reasonable. The Benefit/Risk balance can be considered as each new set of data is collected. Methodologies such as (Quantitative) Structure Activity Relationship, (Q)SAR (including read-across) (e.g., Myatt et al., 2022; 2018), may be useful to collect evidence for early internal decision of the Sponsor. The potential integrative assessment of the evidence in ICH S1B(R1) throughout the drug discovery and development process is illustrated in Figure 4. As discussed earlier, the goal of the WoE assessment is to determine whether a 2-year rat study provides additional value as early as possible during the project so that, if necessary, a late start of the study does not impact the project timeline. To this end, an early start of the chronic rat study might be appropriate for promising projects, to allow for an earlier completion of the WoE assessment. However, in order to minimize animal use on projects that might terminate early, this approach should generally be applied to high priority projects (e.g., expected to enter Phase III clinical trials or have shown early Proof of Concept). Of course, decisions to progress may differ between companies for strategic and scientific reasons. Still, the WoE approach becomes a progressive assessment that collates and absorbs relevant evidence as the project develops; it provides an early decision on whether a rat study is needed or not, and will minimize risk to the project timeline.

### 3.5 Reliability and confidence

Evaluation of the reliability of the data or in general of the acquired information (e.g., available experimental evidence, information from literature), is an essential component of the integrative assessment. Various factors have been suggested for evaluating data reliability (Myatt et al., 2018; Johnson et al., 2022), and these can be taken into account if relevant, including: a) compliance with internationally accepted best practice guidelines; b) agreement with test guidelines; c) data availability for independent inspection; d) concordance with other relevant assessments; e) transparency with respect to deviation from guidelines and protocols as well as discussion of outliers or extreme values (Johnson et al., 2022). In addition to data reliability, it is also critical to evaluate the overall confidence of an assessment (i.e., the strength of the assessment and its uncertainty). Reliability and confidence are different concepts as confidence in the assessment depends on reliability and relevance; relevance of experimental data refers to adequacy for the endpoint and the fit-for-purpose of the test and the corresponding evidence as further discussed by Johnson et al. (2022). The development of a scoring confidence system that can properly grade the different WoE factors is a challenging and complex task. Any assessment, intermediate or final, with a confidence less than high may prompt additional investigations and analysis to strengthen the conclusions. According to Johnson et al. (2022), a high confidence of the assessment suggests that sufficient evidence is available to support an accurate conclusion, and further research is unlikely to increase the confidence.

### 3.6 Metabolites

Consideration should also be given to major human metabolites. Metabolites identified only in human plasma or human metabolites present at greater than 10% of total drug-related exposure that are not present at comparable levels and cannot be qualified by high doses in animal test species, generally require additional safety assessment (FDA, 2020). Therefore, a section describing the metabolic profile and potential carcinogenic risk of major human metabolites is warranted. Discussion of the metabolites for each WoE factor can be incorporated along with the discussions of the parent compound. Various studies (e.g., *in vitro*, short-term dosing major human-specific metabolites) may need to be performed to fill in gaps in the WoE factors for these metabolites.

### 3.7 Reporting

The integrated assessment is to be clearly documented in the final report. A recommended structure of the report is outlined in Section 6. For example, the report would provide both information on timelines and search terms used for a particular search in the case of target biology analysis as well as summary search results. Information derived from toxicity studies will need to be summarized in the WoE assessment with reference links back to the original study reports. In general, the WoE report includes a summary section of each factor complemented with additional details supporting the conclusions in Appendices. A more extended discussion on information gathered for each WoE factor and other supportive information is presented in Sections 4 and 5.

## 4 The six WoE factors

The following sections discuss the elements to be considered when gathering and evaluating evidence from the different WoE areas. The six different WoE factors, as outlined in ICH S1B(R1), are examined below in varying levels of detail depending on how thoroughly the underlying procedures and corresponding best practices are already developed and established. Accordingly, the target biology analysis is presented here in detail highlighting recommended approaches to perform such analysis and gather relevant evidence. The secondary pharmacology WoE factor is discussed in terms of what additional aspects of the standard approaches may be considered to support the ICH S1B(R1) WoE assessment. A similar level of discussion is presented for the histopathology WoE factor from chronic toxicity studies, but it is noted that the guideline already specifies the type of relevant alerting signals that need to be evaluated. The discussion on the genotoxicity WoE factor is brief as the ICH S2(R1) guideline cited in the ICH S1B(R1) addendum fully covers such an assessment. On the other hand, the discussion on hormonal perturbation and immune modulation is hampered by the complexity of the topics. While there are specific examples of hormonal perturbation that are linked to certain carcinogenic outcomes (e.g., estrogen, thyroid hormones), for the majority of cancers these relationships are poorly understood. Similarly, the mechanisms by which effects on the immune system influence human cancer development are still being discerned.

### 4.1 Target biology WoE factor

#### 4.1.1 Background on the target biology factor

The purpose of this individual WoE factor investigation is to determine whether any biological pathways related to the primary pharmacology of the drug candidate (either at the intended tissue site, or as well at other tissue sites where the target may be expressed but therapeutic benefit is not expected) are involved in the development of human cancer. As part of such an assessment, different lines of evidence can be explored, including:

1. Empirical carcinogenicity data on target selective drugs within the same primary pharmacological class. Comparisons to other drugs within a class could (where possible) include an analysis of the similarity of the biological pathways involved, the mechanism of any carcinogenic effects for any previously tested molecules with a positive response in a 2-year rat bioassay (i.e., was the positive result related to target biology or some other factors?), relative potency for any carcinogenic activities related to the primary target for targets with multiple activities, and potentially other aspects such as clinical relevance of the effects, ADME characteristics or considerations based on the principles of read-across (Schultz et al., 2015; 2019).
2. The extent to which the responding biological pathways are well-characterized (e.g., knowledge of the receptor and down-stream or up-stream receptors/genes, interactions with other receptor pathways), and their potential involvement in cancer development (e.g., biological effects of the target exclude a role in immunosuppression, chronic inflammation, oxidative stress, functional interaction with nuclear receptors, and epigenetic effects such as modifications of histones and other structural cellular components). This will also include known human genotypes associated with cancer. Examples of resources to collect such evidence are included in Supplementary Table S1.
3. Relevant carcinogenicity risks related to the pharmacology of any major human metabolites whether related to the intended target of the parent or if there is interaction at closely related isoforms of the target or unintended targets.
4. Any additional links of the target to any of the ICH S1B(R1)-defined WoE factors (e.g., immunosuppression, hormonal effects).

Based on the description of the target biology WoE factor provided in the ICH S1B(R1) addendum, Table 2 outlines several topics to consider in documenting the findings and conclusions pertaining to this area. The outcome of the analysis is any interpretation from the literature/database searches supporting key findings, with the raw results from the literature and database searches included as archived supplementary information.

**TABLE 2:** Outline of the content related to the evaluation of the target biology WoE factor. Notably, evaluation of reliability and potential uncertainties should also be conducted for the data used in the analysis of target biology and primary pharmacology. The detailed report of the target biology analysis is used to draw conclusions on the corresponding WoE factor.

Sections	Description
1. Executive summary	Summary addressing the following points, where appropriate: (1) an evaluation of whether the target biological pathways are well characterized and are demonstrably associated or involved in human cancer development;
	(2) an assessment of any relevant carcinogenicity data available for other chemicals within the same pharmacological class (or absence in the case of first-in-class drugs);
	(3) a carcinogenicity evaluation of major human metabolite(s) and their associated target(s);
	(4) assessment of data reliability and confidence of the analysis with reference for need for further analyses and/or uncertainty clarification;
	(5) a conclusion regarding whether a 2-year rat study would add value to the human carcinogenicity risk assessment.
2. Materials and methods	Description and record of databases examined, literature searches performed and any other data science procedures (e.g., data analysis, artificial intelligence, machine learning, data processing, and modelling).
3. Summary of target pathway(s) and pharmacological class	Background biology information related to normal physiological role of the target pathway and pharmacological class. This could include:
	• summary of the signaling pathways in which the target is involved;
	• cell, tissue, and organ/organ system function;
	• comparison of tissue distribution between species;
	• links to any of the identified WoE factors (e.g., hormonal effects or immune modulation).
	The potential association of target pathways with tumor development would be summarized and assessed for human and target relevance, including examples such as:
	• classification of the target as an oncogene/tumor suppressor or its potential to lead to or exacerbate tumorigenesis;
	• associations made at the pathway level, rather than separately, assessing upstream/downstream pathway components; this analysis would likely involve the interrogation of multiple structured and unstructured (e.g., literature) data sources;
	• use of human genomics databases [e.g., Carss et al. (2023)] to inform wider assessments of target safety, including carcinogenicity risk evaluations;
	• use of gene ontology terms as derived from the database interrogations and mapped onto cancer hallmarks (Chen et al., 2021); hallmarks of cancer represent a conceptual framework that recapitulates the functional capabilities of cells collectively leading to malignant growth (Hanahan and Weinberg, 2000; 2011; Hanahan, 2022);
	• any evidence from the scientific literature and phenotypic databases that directly implicates modulation of target function (such as modulated, hyperactive and hypoactive states) with cancer.
	All of the evidence will be qualified (where appropriate) by species, anatomical location and intervention type.
4. Summary of drug mechanism of action	Information on the pharmacological activity of the drug, and any known human metabolites. This is discussed alongside relevant information regarding the drug class including a description of known/proposed mechanism(s) of action, and a listing of commonly used drug and target synonyms. Also, an assessment can be made of how active the drug is likely to be against rat orthologues, and how this may translate to effective doses in rat and human. Closely related “off target” subtypes (subtypes or isoforms of the primary target) should also be considered when rat carcinogenicity study exposures would be likely to reach pharmacologically active drug concentrations. Relative human/rodent affinities at target exposures at these off-target subtypes in rats and humans can be assessed accordingly to help address human relevance.
5. Carcinogenicity assessment of primary pharmacological class	Discussion on the human relevance of carcinogenicity data for pharmacological class. These data could be obtained from:
	• labels and package inserts obligated by regulatory authorities (noting both the presence or absence of relevant data), and related relevant documentation;
	• published clinical studies including clinical trials and post market surveillance/pharmacovigilance and other human data;
	• published rodent carcinogenicity data including knock-out or other genetically engineered animal models; for example, studies completed by sponsors early in the rasH2Tg model (Sistare et al., 2011; Morton et al., 2014; Hisada et al., 2022) can be helpful for anticipating an association of target modulation with tumor outcome in rodents.
	Additional information, such as the results from (Q)SAR or read-across models (considering substances with the same pharmacology), may be included where they contribute to the mechanistic understanding or support an evaluation of the structural basis of carcinogenicity (or lack of) across chemicals in the drug class.
6. Analysis of cancer risk of major human metabolite(s)	When information on major human-relevant metabolites becomes available, their pharmacological target(s) should be addressed with particular reference to target biology. Carcinogenic potential of such metabolites could be investigated, for example, using (Q)SAR methods. However, an evaluation of secondary pharmacology (e.g., in instances where the principal pharmacological target for a metabolite differs from that of the parent compound) is the subject of WoE factor 2.
	Comparison (e.g., exposure ratios and differences highlighted) of rat and human metabolites could be performed. Results from non-rodent species may be supportive of the assessment of such metabolites.
7. Conclusions	General conclusions drawn based on the topics discussed above reiterating the conclusion from the Executive Summary regarding whether a 2-year rat study would add value to the human carcinogenicity risk assessment.
8. Appendices	Additional information may be gathered including information on:
	• the molecular profile (DNA, RNA and protein structure, binding domains, isoforms, variants, interactions, orthologues, paralogues, degradation, cellular location);
	• anatomical distribution (i.e., a comprehensive review of RNA, protein and operational/functional expression across different cell types, tissues, organs and systems across a range of species);
	Links to archived raw output as as supplementary data file(s) may be provided. Where applicable, a metabolic pathway could be included.
9. Supplementary Information	Raw output from the different literature and database searches can be made available.

Broadly speaking, a 2-year rat bioassay will be considered to add value to the human carcinogenic risk assessment in uncertain situations, when the target biological pathway is either poorly characterized or there are up- or downstream events that are likely to lead to cancer, or the class effects of drugs with activity within this pathway are unknown (or include a risk of cancer). In addition, a first-in-class therapeutic has a higher chance to be considered for carcinogenicity testing unless additional supportive evidence is provided to fill in knowledge gaps to reduce cause for carcinogenic concern for the class. Conversely, if the target is involved in a well-characterized pathway and/or the compound of interest is from a class with well documented effects with positive or negative cancer risk, then it is unlikely that a 2-year rat bioassay will add value.

#### 4.1.2 Target biology WoE evaluation

The target biology evaluation should use a repeatable, transparent, unbiased, and extensive analysis to provide a convincing conclusion regarding the risk of carcinogenicity. This evaluation includes analysis of the literature and relevant biological databases, utilizing similar approaches that have been used for wider assessments of target safety (Brennan, 2017). Integration of data from a variety of genomic and cancer-based resources (examples of which are included in Supplementary Table S1) will inform an assessment of carcinogenic potential (Carss et al., 2023). Emerging approaches such as network biology models may also be considered (e.g., Krämer et al., 2014). Individual literature searches and database queries should be documented, and it is advisable to preserve the unfiltered results. The results should be reviewed for relevance by the domain expert(s) and all key findings discussed to determine whether there is an overall and demonstrable risk of carcinogenicity. Importantly, evaluation of reliability and potential uncertainties should also be conducted for the data used in the analysis of target biology and primary pharmacology.

The report on the target biology analysis should include a balanced integrated evaluation of “negative” findings (i.e., where cancer risks have been investigated and no association with target biology was identified) as well as assessment of the relevance of any potential positive, equivocal, or incomplete information. It is likely that this assessment will broadly cover all aspects of target biology and is performed early in the project timeline [e.g., Target Safety Assessment (TSA)]. A data subset analysis of the main target biology evaluation report(s) used in the WoE assessment would need to focus on carcinogenicity risk endpoints identified in the early target safety assessment. These elements will be extracted into the overall carcinogenicity risk assessment. Notably, the main conclusions from the target biology analysis related to carcinogenicity would be summarized in the WoE report, whereas the corresponding broader, more detailed report can be included in the Appendices of the WoE report, as discussed further below in Section 6.

It should be noted that, although the target biology and primary pharmacology evaluations are needed to support regulatory conversations aligned with ICH S1B(R1), they can also be considered as part of a more proactive strategy started early in the drug discovery and development process (see Figure 4) with initial data (e.g., target biology, genetic toxicity studies) and further data being added to the assessment as it is generated (e.g., histopathology from the chronic toxicology studies is likely the last piece of evidence). Such upfront evaluation, coupled with increasingly informative experimental results from chronic studies, can provide input into product stewardship, and potentially avoid costly and unforeseen impact to the project timeline if a 2-year rat bioassay is determined to be necessary during late-stage clinical trials. Many pharmaceutical companies currently perform a version of this general assessment of target risk (e.g., TSA) either internally or by outsourcing. The TSA could be modified to increase the focus on carcinogenicity endpoints. This early-stage assessment can be used for determining any gaps in carcinogenicity risk assessment which may be filled by incorporation of endpoints into upcoming planned studies or investigational studies [e.g., need for additional nonclinical or clinical data approaches as listed in Figure 2 of ICH S1B(R1)] to minimize the performance of additional studies late in the project.

### 4.2 Secondary pharmacology WoE factor

#### 4.2.1 Background on the secondary pharmacology WoE factor

Documentation of safety risks in humans includes studies of the mode of action and/or effects of a compound not related to the desired therapeutic target. Characterization of the off-target interactions has been termed secondary pharmacology profiling in contrast to primary pharmacology and safety pharmacology studies (ICH S7A, 2000). The safety pharmacodynamic effects of a drug candidate may result from functional interaction with the primary molecular target, secondary targets or non-specific interactions (Valentin and Hammond, 2008).

To investigate the off-target interactions leading to potential safety concerns (secondary pharmacology), industry uses *in vitro* assay panels against multiple unintended targets (i.e., receptors, ion channels, enzymes including kinases, and transporters) with the aim of exploring off-target interactions to focus on selecting more specific molecules to move forward and thus of reducing liabilities potentially leading to toxicity (Valentin et al., 2018; 2023; Jenkinson et al., 2020). The number of targets and target classes tested vary across the industry (Bowes et al., 2012; Bendels et al., 2019; Lynch et al., 2017); however, a trend is emerging with significant overlap in the screening strategies across organizations (Valentin et al., 2018; Jenkinson et al., 2020). The physiological and/or histopathological role of the targets and potential clinical implications usually determine the battery of targets that are selected for the screening. After this, off-target effects are evaluated extensively in *in vivo* regulatory toxicology and safety pharmacology studies.

The guidance for industry on safety pharmacology studies for human pharmaceuticals generally indicates that the design of safety pharmacology studies should consider ligand binding or enzyme assay data suggesting a potential for adverse effects, but it does not recommend the selection of specific targets that should be screened in a secondary pharmacology profiling (ICH S7A, 2000); the only example is the screening against Kv11.1 (i.e., hERG) encapsulated under the ICH S7B (ICH S7B, 2005). As Valentin and Leishman (2023) have recently observed, secondary pharmacology studies are not described in any dedicated guideline but they are sparsely referenced in ICH S7A despite these studies being critical to support hazard identification and human risk assessment, management and mitigation, and they are included in the regulatory submission process together with primary and safety pharmacology studies.

This leads to a potential gap on what targets relative to carcinogenicity assessment are necessary to include in a secondary pharmacology panel to support the discussion on the secondary pharmacology WoE factor. Thus, current panels should be reviewed to ensure that it is clear which targets are relevant to carcinogenicity assessment as it will be discussed further below.

Frequently *in vitro* secondary pharmacology testing is initially conducted at a single concentration, and in such cases the test concentration of 10 µM is used by over 50% of sponsors (Valentin et al., 2018). The 10 µM concentration was historically selected because it offered a >100-fold exposure multiple over the therapeutic free plasma exposure of most small molecule drugs. That said, alternative approaches do exist based on the modalities, the therapeutic, or pharmacological classes and individual organizational strategies. This initial testing narrows the number of targets to be submitted for further evaluation of full concentration-response curves in follow-up functional assay tests. This is required to characterize the drug’s potency, mode of action (e.g., agonist, partial agonist, antagonist) and it also allows to rank compounds of interest with respect to their levels of concern to help guide lead selection. Using this data in conjunction with the drug’s potency on its primary target, an exposure margin at the expected clinical plasma exposure can be estimated for all secondary targets that are suspected to play a role in carcinogenesis. The margin of safety (MOS) is the ratio between the drug’s *in vitro* potency and the unbound clinical plasma concentration; as a rule of thumb, all off-target specific safety margins should typically exceed 30-fold (Redfern et al., 2003; Muller and Milton, 2012; Papoian et al., 2015). In relation to off-target activities, the Cmax (free or unbound) drug concentration is typically used to calculate the MOS. However, the recently released ICH E14/S7B IWG (2022) refers to using both the free and total (i.e., bound) drug concentration especially when species differences in human plasma protein binding (PPB) exist, and for highly PPB drugs. Additionally, the AUC should be considered for MOS determination when appropriate.

#### 4.2.2 Secondary pharmacology WoE evaluation

The secondary pharmacology WoE factor integrates results from off-target profiling for both the specific pharmaceutical being evaluated (see Table 1) and any major human specific metabolites (FDA, 2020). In the context of the ICH S1B(R1) integrated assessment, secondary pharmacology screening is assessed based on promiscuity of the pharmaceutical towards secondary targets (which are not necessarily mechanism-related to cancer). As shown in Table 1, “low target selectivity, off-target activity” is an indication that the 2-year rat carcinogenicity study would add value as compared to “high target selectivity, no off-target activity” (ICH S1B(R1), 2022). As such, a pharmaceutical with a high selectivity and no off-target activity at a large human exposure multiple would provide confidence for a low carcinogenic risk and therefore for a low added value of conducting a 2-year bioassay study. In addition, the ICH S1B(R1) WoE should take into consideration the inclusion of cancer-relevant targets in the secondary pharmacology screen. The screening should evaluate off-target interactions for specific targets “that inform carcinogenic risk (e.g., binding to nuclear receptors)” (ICH S1B(R1), 2022). The ICH S1B(R1) addendum discusses several case studies providing examples of the assessment of secondary pharmacology results (e.g., “No evidence of off-target interactions at drug concentrations up to 10 μM, including no interaction with estrogen, androgen, glucocorticoid receptors”; “Antagonist binding interaction identified for one off-target receptor with Ki 8-fold higher than Cmax at maximum clinical dose”; “Known pharmacology of off-target receptor not associated with tumorigenesis”).

As indicated above, major human specific metabolites should also be evaluated for off-target interactions. Major metabolites currently considered for safety assessment are those identified only in human plasma and present at greater than 10% of total drug-related exposure at steady state (FDA, 2020).

Since most secondary pharmacology targets traditionally tested are human targets, the results are by default of human relevance. However, the sponsor might also consider conducting secondary pharmacology screens on other species-specific or disproportionate metabolites that are evaluated in animal models using a panel of species-specific targets or by means of computational modelling techniques. This might help to shed light into any functional and/or histopathological findings of concern for carcinogenicity that may be species specific, and possibly lacking human relevance.

In the absence of a single “carcinogenicity risk-specific” secondary pharmacology screen, the data from the multiple screens performed during drug development can be summarized for the integrated ICH S1B(R1) summary by pointing out results that inform on cancer risk. For example, no interaction in standard off-target and kinase panels, including binding to pro-inflammatory targets, hormone receptors and/or nuclear receptors, would be relevant outcome generally supporting no value of the 2-year rat bioassay. Insights from secondary pharmacology may be used to explain histopathological findings of concern from the animal models and support the identification and assessment of human-relevant effects (Ribeiro et al., 2020).

Any interaction with secondary targets would prompt an analysis of other supporting evidence assisting an active relationship between such molecular targets and carcinogenesis pathways (including associations with hormonal perturbation and immune modulation that may manifest as histologic findings after 6 months of exposure). An approach like the analysis of the target biology and primary pharmacology mechanism may be envisaged, if necessary, where the human-relevance of any off-target interactions and its possible association with carcinogenicity can be explored. It is also important to remember that secondary pharmacology screens represent human sequence targets, and not those of the rodent, so significant potency differences may exist.

In summary, understanding the characteristics of both on-target and off-target hits through the integrated analysis described above, along with the relative potency and activity compared to the intended target activity at anticipated human exposures, enables development of an integrated risk assessment to further characterize and interpret the functional and/or histopathological findings in animal models and their potential human relevance. Relevant elements useful to summarize the experimental findings from secondary pharmacology results within the ICH S1B(R1) assessment are displayed in Table 3.

**TABLE 3:** Elements to summarize the secondary pharmacology results for each molecular target within the ICH S1B(R1) assessment.

Title	Details
Molecular target	Name of the molecular target (including details such as gene and IUPHAR names and/or Uniprot ID)
Tested chemical	Chemical being tested with indication on whether it is the parent drug or metabolite(s)
Methodology	Short description of methodology including information providing confidence in the assay (e.g., positive and negative controls, number of replicates)
Efficacy	Percentage of maximal response
Potency	*In vitro* binding affinity (IC_50_, K_i_) or cellular functional activity (EC50)
Mode of action	Details on mode of action, e.g., agonist, partial agonist, biased agonist, and antagonist
Human plasma exposure	Cmax and AUC, both total and free
Exposure multiple	Test concentration of drugs/metabolites in relation to the measured or anticipated clinical exposure (e.g., 10-, 30-, 100-, 300-, and/or 1000-fold multiples)
Margin of safety	Assessment of *in vitro* off-target potency in relation to human exposure (e.g., the ratio between the *in vitro* activity and the unbound clinical plasma concentration)
Likelihood of carcinogenic risk to humans with evaluation of the confidence	Conclusion on carcinogenic risk to humans

Abbreviations: AUC: the area under the plot of plasma concentration of drug against time after drug administration; Cmax: the maximum or “peak” concentration of a drug observed after its administration; EC_50_: half-maximal effective concentration; IC_50_: half-maximal inhibitory concentration; IUPHAR: International Union of Basic and Clinical Pharmacology (IUPHAR/BPS, 2023); K_i_: inhibition constant; UniProt: universal Protein Resource (UniProt, 2023).

#### 4.2.3 Cancer-related off-targets panels

The addendum emphasizes the importance of targets that inform carcinogenic risk such as binding to nuclear receptors (Table 1). In general, several targets, such as aryl hydrocarbon receptor (AhR) (Murray et al., 2014), p38 kinase (Kudaravalli et al., 2022) or epigenetic targets (Herceg et al., 2013), have a demonstrated role in development of some types of tumors, but a full comprehensive list of targets critically associated with a carcinogenic risk has not been identified. Screening panels specifically including cancer-related targets are being proposed, where to our knowledge, the scientific rationale on the association between the targets and carcinogenic potential has not been fully elucidated (Eurofins, 2023). Comprehensive literature searches based on cancer-gene databases might support the identification of cancer-relevant targets and currently activities are under way to isolate, review, and describe targets (Rider, 2023) that might then be used as biomarkers in assessing the carcinogenic potential of chemicals. When associations are identified, further investigation of available evidence is needed to demonstrate the causal relationship between a given target and cancer, as well as its human-relevance.

As summarized by Tice et al. (2021), numerous targets can be involved in carcinogenesis, e.g., activation of PI3/AKT signaling through G protein-coupled receptors (GPCRs) and receptor tyrosine kinases (Martini et al., 2014). Carcinogens may act through modulation of receptor-mediated effects (e.g., estrogen receptor (ER), peroxisome proliferator-activated receptor (PPAR), and AhR) or modulation of endogenous ligands (including hormones) (Smith et al., 2016; 2020). Attention has been devoted to nuclear receptors (Dhiman et al., 2018; Zhao et al., 2019) and their co-regulators (Lonard and O’Malley, 2012) that play crucial roles in normal physiological processes, and alterations of such receptors impact the development of cancer. Examples of nuclear receptors’ involvement in cancer are hormone-dependent cancers (e.g., estrogen-dependent breast cancer) (Emons, 2022). There is a considerable overlap between the processes involved in receptor-mediated effect modulation and hormonal effects given the involvement of receptor-based signaling in both cancer and endocrine disruption. Receptors involved in receptor-mediated rodent carcinogenesis include constitutive androstane receptor (CAR), PPAR alpha, and AhR (Klaassen, 2019).

Notably, some targets that are usually employed in secondary pharmacology screening (Bowes et al., 2012; Lynch et al., 2017) are associated with cancer-related AOPs as derived from the AOP wiki (AOP Knowledgebase, 2023); these targets are, for example, AR Human Androgen nuclear hormone receptor (NHR), D2S Human Dopamine GPCR, Beta-2 Human Adrenoceptor GPCR, and Human PPAR gamma NHR. The off-target panels described by Bowes et al. (2012) and Lynch et al. (2017) also include several targets associated with immune effects (e.g., Cannabinoid receptor CB_2_, Lymphocyte-specific protein tyrosine kinase, Adenosine A_2B_ Receptor) and endocrine effects (e.g., Dopamine receptor D_2_ and Serotonin 1A receptor 5-HT_1A_). The effects associated with a given target are specifically reported by Bowes et al. (2012) and Lynch et al. (2017) as derived from the analysis of adverse drug reactions (ADRs) described in the literature.

The development of a cancer-related off-target panel would need to pay special attention to the human relevance of the pathways underlying a specific off-target activity. For example, Beta-2 Human Adrenoceptor GPCR is associated with the AOP involving Beta-2 adrenergic agonist activity leading to mesovarian leiomyomas in the rat and mouse, but this pathway is considered human irrelevant by the scientific community (Kelly et al., 1993; ECETOC, 2006). On the other hand, the human relevance of anti-dopaminergic activity (D2S Human Dopamine GPCR) leading to mammary adenomas and carcinomas in the Sprague-Dawley rat is still controversial (Harvey, 2005). Additionally, the relationship between targets and AOPs should be ultimately evaluated in terms of relevance to clinical use according to the elements in Table 3.

A cross sector effort involving safety scientists from academia, industry, service and technology providers and health authorities should be established to support the development of a cancer-related panel of targets to support the ICH S1B(R1) secondary pharmacology factor. Similar initiatives have led to the successful identification of targets associated with key safety risks as in the case of seizure liability (Easter et al., 2009; Roberts et al., 2021).

### 4.3 Histopathology from chronic studies WoE factor

Histopathology evaluation of toxicology studies, especially chronic toxicology studies, may identify proliferative or pre-neoplastic lesions as specified in the ICHS1B(R1) histopathology WoE category. These lesions may also provide information that contributes to the assessment of other WoE categories, including hormonal effects and immune modulation. Lesions that may be expected from the targeted pharmacology, or the secondary pharmacology that are described in the earlier sections of the WoE, may also be observed in the chronic toxicology study histopathology.

The presence or lack of proliferative or pre-neoplastic changes in the chronic toxicology studies is certainly an important factor in the WoE evaluation. When proliferative or pre-neoplastic changes are identified, the pathologist or toxicologist is left with interpreting the relevance or non-relevance of the findings to humans. Rodent specific findings considered not relevant to humans have been described and are documented in the public literature. Findings of unknown clinical significance will shift the WoE assessment to identifying that additional investigative studies may be needed and/or that a 2-year rat study may add value to the carcinogenicity risk assessment. The ICH S1B(R1) addendum provides a detailed description of relevant histology findings from chronic studies that would be considered alerts for carcinogenic potential. The 6-month rat study is expected to be the main source of information but other types of studies (shorter-term rat studies, longer-term non-rodent studies, longer-term mouse studies, and early clinical data) can be integrated to build the WoE assessment or provide earlier alerts to potential carcinogenic risk.

The original description of the preneoplastic constellation of observations (e.g., cellular hypertrophy, cellular hyperplasia, persistent tissue injury and/or chronic inflammation, foci of cellular alteration, preneoplastic changes, and tumors) gathered from repeated-dose toxicity studies (with emphasis on the 6-month rat study) is reported in Table 1. The full pathology report and individual animal findings should be examined for proliferative findings that may not be highlighted in the main summary. It should be noted that standard terminology for cancer-relevant histopathological findings should be utilized in study reports and histopathology interpretations. An example of this terminology is the INHAND criteria (www.goreni.org). Participation of an expert pathologist in this part of the WoE evaluation is necessary.

The evaluation of this WoE factor should include presentation and discussion of the plasma exposure margin of the parent and any major metabolites relative to clinical exposure. The dose corresponding to the plasma exposure at which pre-neoplastic effects are observed from animal studies (and if it is dose-dependent) can be extrapolated to a human equivalent dose (HED) in the early phases of the WoE evaluation or, if human exposures are known, animal exposures can be directly compared to the human AUC or C_max_, as appropriate. The occurrence of proliferative findings at a high exposure multiple that will not be reached in the clinic could mitigate the need for a 2-year rat study when the WoE data are integrated. This potential human exposure risk relative to exposures in animal studies is used as part of the overall WoE assessment.

It is important to discuss the relevance of rodent lesions (proliferative and non-neoplastic) that occur with an incidence level above study matched controls or appropriate historical controls. Spontaneous genetic alterations occur in commonly used rodent strains, and genetic drift should be considered if unexpected findings occur when changing animal suppliers or test facilities. Also, especially as new mouse models of disease are investigated, unexpected histologic pre-neoplastic findings may be observed and must be interpreted in conjunction with mouse genetics and strain background (e.g., Alison et al., 1994; Szymanska et al., 2014). An example of a rodent-specific finding is the induction of alpha 2u-globulin nephropathy in male rats, which has data to support that it is not relevant in human risk assessment (Swenberg, 1993). The goal of investigative studies would be to increase the understanding of the relevance of changes present in toxicology studies to humans, potentially due to differences in anatomy/physiology, metabolism or because of differences in sensitivity, with human exposure being below the threshold at which homeostasis is perturbed. Overall, understanding of the pathogenesis of the lesions and the underlying mechanism would support the evaluation of human relevance as well as the WoE integrated assessment.

As regards to mechanistic interpretation, chronic inflammation, for example, creates a local microenvironment that can induce genomic instability in cells (Smith et al., 2016; 2020; Tice et al., 2021). Inflammation generates various mediators including cytokines, reactive oxygen and nitrogen species (ROS and RNS respectively), serine and cysteine proteases, membrane perforating agents, matrix metalloproteinase (MMP), tumor necrosis factor alpha (TNFα), interleukins (IL-11, IL-6, and IL-8), interferons (IFNs) and enzymes, as cyclooxygenase-2 (COX-2), lipooxygenase-5 (LOX-5) and phospholipase A2 (PLA2), which activate or are activated by transcription factors such as nuclear factor-κB (NF-κB) and signal transducers and activators of transcription-3 (STAT3) (Vendramini-Costa and Carvalho, 2012). These events induce oxidative stress and facilitate mutations, epigenetic changes, or genomic instability (Multhoff et al., 2012; Vendramini-Costa and Carvalho, 2012; Wu et al., 2014; Ding et al., 2019) while prolonged release of the inflammatory mediators facilitates growth, progression, and tumor invasion. Potential investigative studies that examine the key elements of chronic inflammation could serve as additional data for the overall WoE.

### 4.4 Genotoxicity WoE factor

Genetic toxicology testing assesses whether a compound can cause DNA damage that leads to heritable defects and thus potentially cancer. There is abundant evidence that genetic alterations constitute a cancer risk and may be a prerequisite to tumor development. Thus, genetic toxicology assessment has been a standard for evaluation of cancer risk for many decades. In the drug discovery and development process, the genotoxicity potential of a drug candidate is assessed by means of a series of genetic toxicity tests according to a core battery well defined by the regulatory guideline ICH S2(R1) (2012). ICH S2(R1) should be used in conjunction with ICH S1B(R1) for understanding the interpretation of the results of the genotoxicity battery for the WoE determination. Unequivocally negative (or resolved positive or equivocal findings resulting in a WoE conclusion that genetic toxicity is of low risk) or positive genetic toxicity results as defined by ICH S1B(R1) provide evidence that a 2-year rat bioassay is less likely to add value to the carcinogenicity risk assessment. Alternatively, genetic toxicity results that are of uncertain relevance to humans (which cannot be resolved by investigative approaches described in relevant guidelines) will indicate that a 2-year rat bioassay will add value to the human carcinogenicity risk assessment.

The ICH S2(R1) core battery includes two options. In option 1, *in vitro* tests (a bacterial reverse mutation assay and a cytogenetic test for chromosomal damage or a mouse lymphoma *Tk* gene mutation assay) are conducted to evaluate gene mutations and chromosomal damage followed by an *in vivo* evaluation of chromosome level effects. Additional *in vivo* tests may be needed as a follow-up strategy for positive or equivocal results in option 1. The option 2 battery includes the *in vitro* bacterial reverse mutation assay and *in vivo* testing of two genotoxic endpoints in two tissues. Other tests that are conducted in addition to the ICH S2(R1) core battery to investigate the genotoxicity mechanisms and the relevance of the response to humans (as appropriate) are, for example, (Nicolette, 2017): a) *in vitro* comet or alkaline elution (different cell types) conducted as early screening and for mechanistic evaluations; b) *in vivo* comet conducted to further investigate positive bacterial or mammalian *in vitro* tests from the core battery; c) transgenic rodent gene mutation to further investigate *in vitro* gene mutation results; d) mammalian Erythrocyte Pig-a Gene Mutation Assay particularly following Ames positive results (Robison et al., 2021). Further reading on the combination of genotoxicity results for genotoxicity assessment is in the publication by Hasselgren et al. (2019).

### 4.5 Hormonal perturbation WoE factor

**FIGURE 5:**
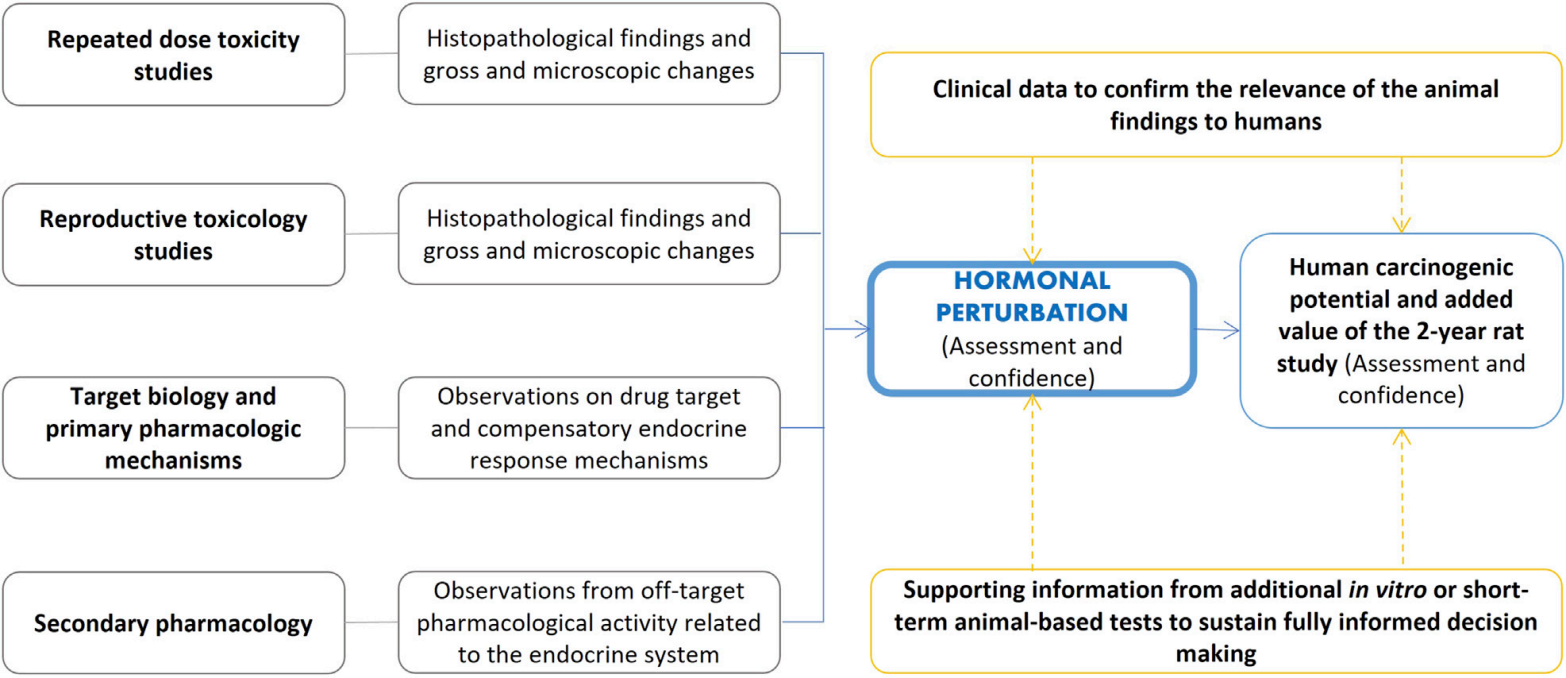
Assessment of hormonal perturbation contributing to the evaluation of human carcinogenic potential and the added value of the 2-year rat study. The ICH S1B(R1) addendum refers to relevant signals from repeated-dose toxicity studies and reproductive toxicology studies (including, for example, changes in organ weights, see Table 1). Secondary pharmacology screens may also inform on the interactions with targets associated with the endocrine system. Supporting evidence from other investigative approaches may aid intelligent decision making.

The evaluation of hormonal effects potentially leading to carcinogenic risk is a critical component of the weight of the evidence evaluation originating from different sources as outlined in the ICH S1B(R1) addendum (see Table 1). This assessment is illustrated in Figure 5. The evaluation of hormonal perturbation is primarily based on findings from repeated-dose toxicity studies and relevant signals from reproductive toxicology studies that suggest hormonal perturbation. These include microscopic changes in endocrine or reproductive tissues of atrophy, hypertrophy, and hyperplasia and/or biologically significant endocrine and reproductive organ weight changes which are not explained as findings secondary to processes such as stress or altered body weight (ICH S1B(R1), 2022). If there is concern for potential endocrine effects early in the development program, hormonal measurements can be made during the 4-week or 6-month toxicology studies and results compared to clinical data to assess the relevance to patients. Alternatively, targeted hormonal studies can be conducted once a specific concern is identified. In designing these studies, care must be taken to ensure that samples are taken at appropriate time points to minimize impact of diurnal or reproductive cycles on the results.

As outlined by the guideline, knowledge of drug target and compensatory endocrine response mechanisms is also an element to consider, and this knowledge can be acquired within the analysis of the target biology WoE factor. Notably, secondary pharmacology screening may inform on potential interactions with targets that have been associated with the endocrine system (Bowes et al., 2012; Lynch et al., 2017). Additionally, investigative approaches (e.g., *in vitro* studies with cells from endocrine-controlled tissues) may help to clarify potential concerns. Moreover, confirmation of hormonal changes identified in animal studies with samples taken in clinical trials may confirm the relevance of the animal findings to humans.

As mentioned earlier, it is essential to understand pathogenesis and human relevance of hormonal perturbations. This would also include discussion of the plasma exposure margins.

### 4.6 Immune modulation WoE

#### 4.6.1 Immune modulation WoE assessment

The WoE integrated assessment requires the evaluation of the immune modulation factor according to the ICH S8 guideline, which applies to new human pharmaceuticals (ICH S8, 2006). The ICH S8 guideline restricts immunotoxicity to “unintended immunosuppression and immunoenhancement, excluding allergenicity or drug specific autoimmunity”. Evaluation of immune modulation is based on a weight of evidence that requires additional immunotoxicity testing based on the following constellation of observations (a single positive signal prompts additional in-depth studies on the potential concern for immunotoxicity):

• Preliminary toxicology findings indicating immune modulation from standard toxicity studies (rodent and non-rodent studies from early short term to more chronic repeated-dose studies); the ICH S8 guideline lists the relevant signals indicating potential immunosuppression or enhanced activation of the immune system.
• Pharmacological properties of the compound that indicate potential modulation of the immune function.
• The intended indication and patient population to evaluate whether the intended patient population is already in an immunocompromised state.
• Structural similarities to known immunomodulators.
• Disposition properties of the drug to evaluate whether the drug is retained at high concentrations in cells of the immune system.
• Clinical observations in case of on-going clinical trials.

**FIGURE 6:**
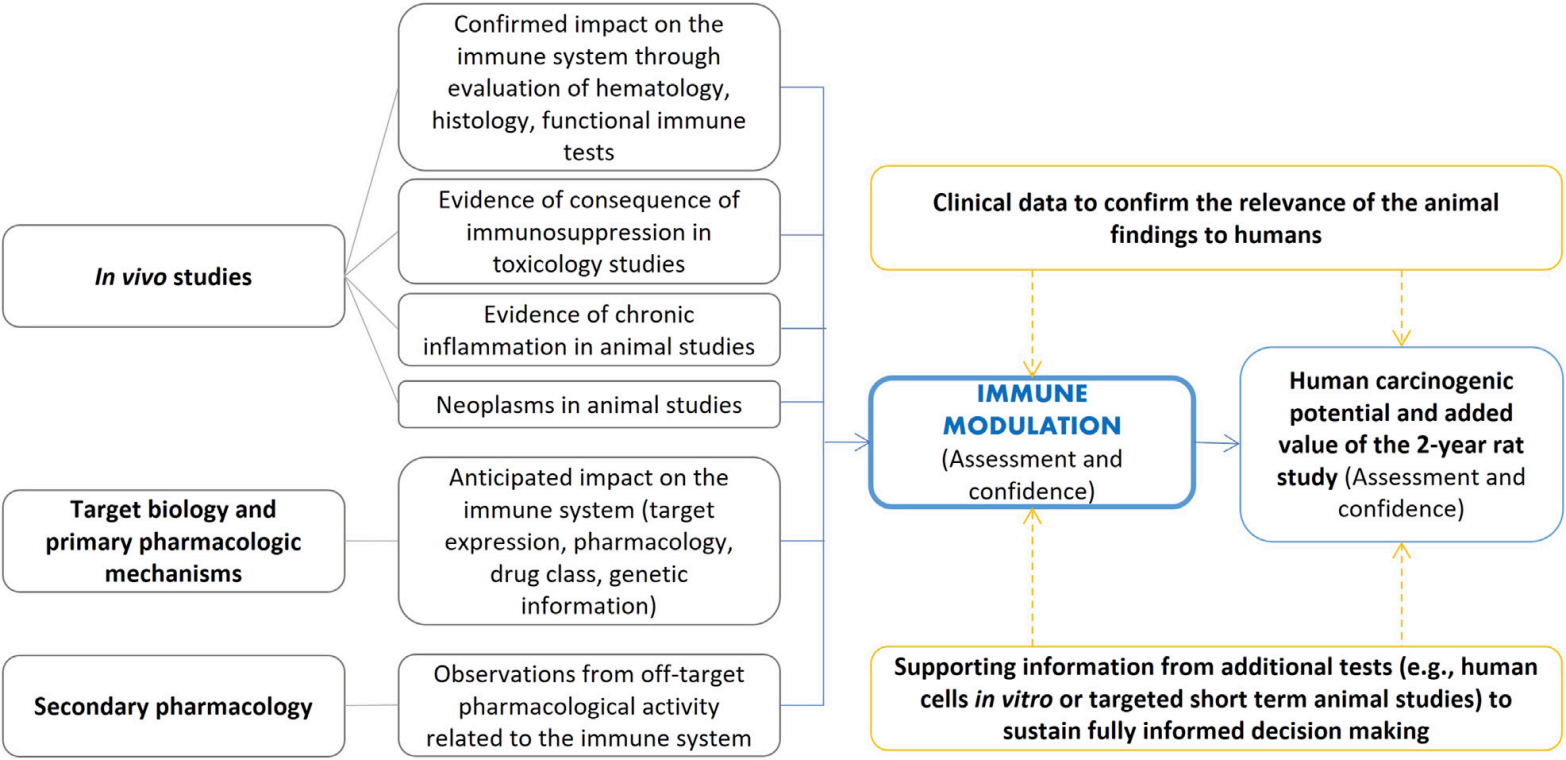
Examples of elements that can inform cancer risk assessment for immunomodulators as adapted from Lebrec et al. (2016) and framed in the ICH S1B(R1) assessment.

The new FDA guidance on Nonclinical Evaluation of the Immunotoxic Potential of Pharmaceuticals (FDA, 2023b) provides additional information on assessment of immune function relating to carcinogenicity specifically noting the need to consider the potential for a drug candidate to increase tumor promotion, growth, and metastasis. Additional points of consideration include “effects of the pharmaceutical on key immune components thought to be involved in tumor surveillance (e.g., NK cells, T cells, antigen-presenting cells), such as downregulation or functional impairment of key immune-cell populations” (FDA, 2023b). Figure 6 summarizes examples of elements that can inform cancer risk assessment for immunomodulators (Lebrec et al., 2016) framed into the ICH S1B(R1) assessment.

#### 4.6.2 Immunosuppression

Several carcinogens can act largely via immunosuppression and this is particularly true of drugs intended to prevent transplant rejection [e.g., cyclosporin (Rafferty et al., 2012)] and some classes of agents intended to treat inflammatory diseases. Immunosuppression may not directly transform normal cells into potential tumor cells. Instead, immunosuppression can both inhibit and potentiate neoplasia with pre-neoplastic cells that manage to evade mechanisms of elimination thereby having their survival and/or replication facilitated (Bugelski et al., 2010; Lebrec et al., 2016; Smith et al., 2016).

The relationship between the immune system and development of cancer (Lebrec et al., 2016; Ponce, 2018) has been related to different mechanisms including tumor immunoediting (Dunn et al., 2002), oncogenic viruses (Engels et al., 2008), chronic inflammation (Mantovani et al., 2008), and chronic B cell stimulation (Küppers, 2005). Though not true for all cases, many of the cancers known to be associated with chronic immunosuppression (e.g., transplant recipients, HIV/AIDS) appear to be related to chronic infection (e.g., viruses, bacteria, parasites). Each of these mechanisms can occur simultaneously (Ponce, 2018).

An FDA and HESI funded workshop (Lebrec et al., 2016) concluded there is a limited understanding of the quantitative relationship between immunosuppression and cancer risk, stating that an increased focus on new approaches for monitoring immune function and early detection of cancer risk in humans is needed. Information from nonclinical experiments, clinical epidemiology and immunomodulatory therapeutics show that the complex link between immunosuppression and cancer risk is multifactorial and does not correlate well with the 2-year rodent bioassay (Bugelski et al., 2010; Lebrec et al., 2016). This view is supported by ICH S1B(R1) and the recent FDA guidance (FDA, 2023b) which notes that “animal models, including rodent carcinogenicity studies, have been shown to be of limited help in identifying an increased cancer risk that may arise in patients as a consequence of immunosuppression”, an observation that is “particularly true when the increased tumor risk is caused by recrudescence of latent viral oncogenes, infectious agents, or chronic inflammatory states, for which significant species differences exist that make clinical translatability challenging”. Furthermore, cancer risk associated with immunosuppression cannot be assumed to be similar for all immunomodulatory molecules. Any evaluation therefore needs to be a mechanism-based weight-of-evidence approach, including data from immune function tests and their relationship to tumor initiation, immunosurveillance, and tumor promotion, in addition to the consideration of underlying human disease (ICH S8, 2006; Lebrec et al., 2016; FDA, 2023b). Of interest, the lack of human predictivity in rodents may be related to differences in structure, development and function of the immune system between rodents and humans (Haley, 2003; Holsapple et al., 2003; Kotturi et al., 2009; Bugelski et al., 2010). The use of human cells *in vitro*/*ex vivo* to screen for potential immunomodulatory effects has demonstrated encouraging potential (Phadnis-Moghe and Kaminski, 2017) and may serve to augment current immunologic investigations.

As noted in the ICH S1B(R1) guideline, a 2-year rat study is less likely to add value when there are either no effects on the immune system (e.g., in a 6-month rat or 9-month non-rodent study) or when broad immunosuppression is expected based on target biology evaluation or results of standard toxicology studies and immunotoxicity follow-up testing (as recommended by ICH S8). In the latter case, while a human carcinogenicity risk is expected, this can be addressed by appropriate discussion in the WoE document and product labeling. Findings of tumors in clinical trials of immunosuppressive agents will guide stricter labeling (e.g., boxed warning). Assessment of the impact of immunosuppressive or immunomodulatory activity on carcinogenic risk is expected to gain no further insights from the conduct of a 2-year rat study.

## 5 Other information

### 5.1 Additional studies

It is expected that additional studies including novel technologies that target identified knowledge gaps in the WoE assessment and support the understanding of human relevance of signals, could complement the evidence from the six WoE factors. These would help to clarify potential concerns and aid intelligent decisions. In general, any novel investigative approach that is based on rigorous scientific methods may provide useful evidence. An example of this may be the quantitation of clones with cancer driver mutations (Marchetti et al., 2023). Importantly, attention should be paid to the quality of conduct of these studies and how widely accepted the proposed studies are (scientifically and by regulatory agencies).

The ICH S1B(R1) mentions (but not limited to):

• Nonclinical approaches: special histochemical stains, molecular biomarkers, serum hormone levels, immune cell function, *in vitro* or *in vivo* test systems, data from emerging technologies.
• Clinical approaches: generated to inform human mechanistic relevance at therapeutic doses and exposures (e.g., drug concentrations in urine and evidence of crystal formation; targeted measurements of clinical plasma hormonal alterations; human imaging data).

### 5.2 *In silico* approaches

For the assessment of complex endpoints, there are known issues and limitations to employing *in silico* approaches including (Q)SARs in isolation; however, their use within an integrated assessment framework to help explain specific experimental signals, is justified.

For example, while not routinely performed, application of appropriate *in silico* methods can support secondary pharmacology screening to fill in data gaps in experimental profiling (Jenkinson et al., 2020) and they may become more commonplace in the future. Experimental screening can be combined with predictions from computational models (e.g., statistical- or expert-based systems) if they are developed using an adequate experimental dataset for cancer-related targets and covering an appropriate chemical space. However, such models must be used with caution to avoid the problem of unpredictable events, and furthermore are not a prerequisite but only one tool to aid the expert judgement.

Moreover, *in silico* approaches can make use of resources that collect carcinogenicity study findings with details on the histopathological findings from the corresponding animal studies. Various publications have reviewed the carcinogenicity databases together with available (Q)SAR models that are based on such databases (Benigni et al., 2008; Golbamaki and Benfenati, 2016; Bossa et al., 2018; Bower et al., 2020). Additionally, various platforms are available to search these databases and/or run the (Q)SAR models [e.g., (Myatt et al., 2017; Roncaglioni et al., 2022; LCDB, 2023)]. Notably, *in silico* models are also being discussed to predict the human carcinogenic potential based on relevant PubChem bioassays (Chung et al., 2023). The Cancer Potency Database (CPDB) is a key repository of chronic, long-term animal cancer bioassays (Gold et al., 1984; Gold et al., 2005) that classifies chemicals based on multiple-organ toxicity data. It provides access (NIH, 2023; Instem, 2023; LCDB, 2023) to several other data sources including histopathological findings on neoplastic and non-neoplastic lesions, such as those described in the NTP reports of short-term toxicity and long-term carcinogenicity (CEBS, 2020). CPDB has been serving as the basis for the development of several *in silico* models, including organ-specific (Q)SARs (Lagunin et al., 2018). To facilitate the construction of such organ-specific carcinogenicity models, the CPDB has been used by FDA to develop a liver cancer specific database (Young et al., 2004).

A repository of data from 2-year rodent bioassays is also maintained by FDA’s Center for Drug Evaluation and Research (CDER) (Matthews and Contrera, 1998; Bourcier et al., 2015) and it has been used to develop *in silico* models (e.g., Matthews et al., 2008; Kruhlak et al., 2015; Guo et al., 2017).

The EPA Toxicity Reference Database (ToxRefDB) is an example of repository where chemicals are classified as positive or negative for preneoplastic or neoplastic lesions in rat and mouse for multiple tissues (Watford et al., 2019).

The Registry of Toxic Effects of Chemical Substances (RTECS) [initially maintained by US National Institute for Occupational Safety and Health (NIOSH)] is a database which collects tumorigenic dose data from positive or equivocal tumorigenic reports and affected organ, tissue or functional systems; RTECS classifies the test-compounds as carcinogenic, neoplastic (evidence for tumors lacking invasiveness but that could not definitely be classified as either benign or malignant), or equivocal (NIOSH, 1997).

The application of read-across supported by the use of *in silico* techniques, can be useful within the WoE assessment framework. This approach aids the examination of similarities and differences between a data-poor substance (the target chemical) and a chemically similar data-rich substance. Overall, the use of computational models such as artificial intelligence (AI), expert systems, statistical machine learning methods like QSARs and emerging methodologies could be considered in the context of fit-for-purpose evaluations to be added to the integrated WoE assessments. AI may become an increasingly valuable asset in the future (Hartung, 2023). Today, *in silico* or computational methods can provide screening, targeted read-across, review of similar analogues and identification of areas of concern or toxicophores in a target compound. However, to avoid generation of unnecessary data and potential false (positive or negative) results, such models should be used in a judicious and targeted manner and not in isolation. Several considerations must be evaluated when selecting models including, for example, training set breadth, endpoints, model performance, validation and applicability domain. Expert judgement can guide such selection and interpretation. However, as the technology exists today, the use and application of computational methods should be carefully considered and the results evaluated and integrated alongside the other considerations outlined herein.

### 5.3 First-in-class

First-in-class drugs, those as defined by the FDA that “have mechanisms of action different from those of existing therapies” (FDA, 2023a), may require particular attention and review under the ICH S1B(R1) framework. For novel drug targets, the integrative WoE assessment is still considered eligible, though higher evidentiary standard to compensate for the lack of precedent experience with the drug target would be required to demonstrate no cause for concern.

In such cases, the target biology analysis may still be used to demonstrate with strong evidence that target biology is not associated with cancer development showing that the pharmacology and pathways are sufficiently well-characterized and no plausible links to cancer development related to the primary pharmacology biological pathways are identified (the best example would be a non-mammalian target). A lack of proliferative changes or tumor signal in any organs/tissues should be demonstrated at a high multiple of exposure in the 6-month rat study (or pharmacologically relevant species, such as the 9-month non-rodent). In such situations where this may be questionable, it may be prudent to generate additional supporting evidence (e.g., special histochemical stains, molecular biomarkers, serum hormone levels, data from emerging technologies, or immune cell function integrated into the 6-month rat study) and/or compare the No Observed Adverse Effect Levels (NOAELs) from the 1-, 3- and 6-month rat studies taking into account that exposure margins may change with an increase in the duration of exposure. Collaborative initiatives (e.g. (Corton et al., 2022)) have been launched to investigate the value of emerging technologies that may provide such a higher evidentiary standard. Sponsors can apply customized and creative investigative approaches that could address the uncertainty or inform human relevance of the identified risk. Clinical data generated to inform human mechanistic relevance at therapeutic doses and exposures may provide potential evidence. In addition, data from longer-term toxicity studies in non-rodents and mice may also be instrumental in providing additional information on the human relevance of rat study findings (i.e., demonstrating that the rat study findings are species specific).

When the results from the rasH2-Tg mouse study are available, they should be included in the WoE document and a negative result can contribute with other available evidence to further derisk first-in-class drugs when pharmacologic target engagement can be demonstrated in the rasH2-Tg model.

## 6 Suggested WoE report structure

The WoE integrated carcinogenicity risk assessment addresses the six WoE factors (as noted in the above sections) and could include considerations of metabolites, evidence from additional special studies and clinical data coupled with the integrated assessment according to the following suggested table of contents:

• Executive summary that summarizes the integrated assessment
• Target biology
• Metabolite profile and ADME
• Secondary pharmacology
• Genetic toxicity
• Histopathological findings in chronic toxicity studies
• Hormonal perturbation
• Immune modulation
• Additional special studies
• Clinical data
• Guidance/Advice from other regulatory authorities (if any)
• Data integration and human relevance including overall conclusions
• Appendices

The different sections summarize the findings and relevant conclusions for the integrated assessment whereas additional details of the assessments can be included in the Appendices. Summary tables may be included for each WoE factor reporting information such as the types of studies (e.g., human, animal, and *in vitro*), strengths/limitations of evidence from each study (if applicable), confidence in the outcomes for each study and any other data considered (e.g., ADME and clinical data). The evidence assessment of each study should address the relevance of the *in vitro* or *in vivo* findings to a biologically plausible mechanism in humans.

**FIGURE 7:**
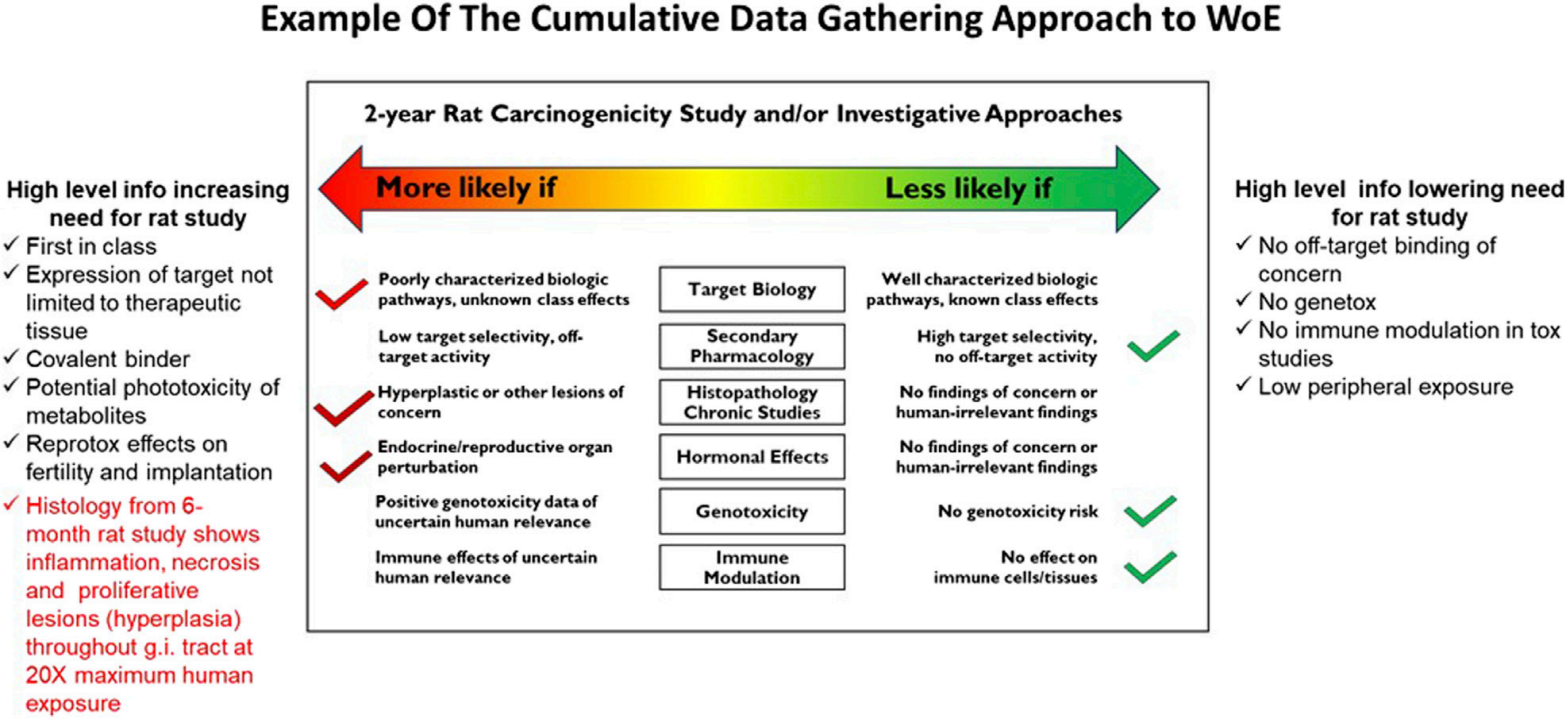
The format that can be used to summarize relevant evidence and corresponding conclusions. The core image is originally taken from the ICH S1B(R1) guideline, and it can be updated with relevant evidence as soon as it becomes available. Notably, the 2-year rat bioassay is less likely to be of value also in the case of evidence of unequivocal genotoxicity or broad immunosuppression indicating a carcinogenic risk to humans (ICH S1B(R1), 2022). This figure is a “living” sliding scale to be updated at each stage gate. In this example, the 6-month rat study histology confirms lesions consistent with carcinogenic risk and may be used as the critical information to spur a decision on the need for a 2-year rat carcinogenicity study. The results from the 1-month or 3-month studies can also be useful to get an early indication of a problem, but if negative they will not be definitive.

A final table in the “Data integration and human relevance including overall conclusions” can then condense the conclusions and confidence from the WoE factor tables. Overall strength of evidence from each WoE factor and human relevance conclusions provides the overall rationale in support of the integrated assessment conclusion of whether or not a 2-year rat bioassay will add value to the human cancer risk assessment. This summary table can work in concert with the visualization provided in Figure 7 where each factor can be commented in relation to the overall balance of data towards the WoE assessment.

Figure 7 can also serve as a “living” sliding scale to be updated during the project timeline. Applying the data to the summary table and to this figure and adding new data from subsequent studies as they become available, can help identify gaps in information that might need special assessment in upcoming studies (e.g., clinical data or other assessments of human relevance or histology endpoints in a repeat dose toxicity study) and track whether knowledge gaps have been filled. Figure 7 exemplifies the cumulative data gathering approach to the WoE integrated assessment. The use of this type of approach can aid in making an early decision as to which of the three WoE outcomes is expected (carcinogenic potential in humans is likely, unlikely or uncertain) and to evaluate whether a 2-year rat study would add value to the human carcinogenicity risk assessment. This will allow for a timely decision to begin the activities on running a 2-year rat bioassay to be made with minimal impact to the project timeline.

## 7 Discussion

The current work presents a procedural framework that helps develop and apply the WoE integrated approach to support a derivation of a scientifically-sound opinion on whether the 2-year rat study provides relevant additional information on carcinogenic risk to humans. Experts from multiple organizations have collaborated to propose a transparent and pragmatic consensus procedure supporting the ICH S1B(R1) WoE carcinogenicity assessment. First, this paper presents each of six WoE factors and describes how these factors contribute to add evidence for the overall WoE assessment. These factors are discussed with varying degrees of thoroughness, reflecting the current development and best practices associated with the evaluation of each factor. Second, the proposed procedure recommends an organized timely approach to data collection that highlights the importance of transparency in presenting the data and how the data itself is collected, and it advocates the evaluation of data reliability and the estimation of confidence in the assessments leading to the final outcome. The six Weight of Evidence (WoE) factors, as outlined within the ICH S1B(R1) guidelines, can be conceptualized as interconnected components within a comprehensive assessment framework, collaboratively employed to scrutinize and elucidate observed signals (or the lack thereof). Cross-integration of evidence from the different factors leads to a network of evidence for critical discussion and presentation of a structured WoE document. The systematic approach presented here also includes a framework for preparing the carcinogenicity risk assessment document both for presentation to the regulatory authorities or for internal use.

The progressive nature of the integrative WoE carcinogenicity assessment adopted by sponsors over the course of their own development programs, encourages addition of new evidence as it becomes available. In general, this progressive approach, is a critical process to reach an early conclusion on the added value and need of the 2-year rat carcinogenicity study thereby enabling timely product stewardship.

The application of the procedural framework proposed herein is expected to consistently support application of the scientifically-based integrated approach and to increasingly promote the successful implementation of the WoE approach to carcinogenicity assessment and further the elimination of unnecessary animal studies by reduction of the need to conduct the 2-year rat carcinogenicity study. Even if a 2-year rat study is ultimately required, creation of a WoE assessment is valuable in understanding the specific factors and levels of human carcinogenic risk better than have been identified previously.
