# Supplementary material for: Developing a pragmatic consensus procedure supporting the ICH S1B(R1) weight of evidence carcinogenicity assessment
Source: Front Toxicol. 2024 Apr 5;6:1370045. doi: 10.3389/ftox.2024.1370045 (PMC11027748; doi:10.3389/ftox.2024.1370045)
Supplement: Supplementary file 1 [file Table1.pdf]

SUPPLEMENTARY MATERIAL

Table S1. Examples of public resources available to support the assessment of the target biology and primary pharmacologic mechanism WoE.

**Resource**	**Examples of relevant data**
PubMed (https://pubmed.ncbi.nlm.nih.gov)	Biomedical literature citations from MEDLINE, life science journals, and online books.
eTRANSAFE (https://etransafe.eu/)	Translational safety data.
ChEMBL (https://www.ebi.ac.uk/chembl/)	Target pharmacology
ClinicalTrials.gov (https://clinicaltrials.gov/)	Adverse events in clinical trials
Ensembl (https://ensembl.org)	Phenotypes, traits and disease associations, across multiple species, many relating to cancer.
DepMap (https://depmap.org)	Genetic and pharmacological dependencies in cancer cell models.
Cancer Gene Census – COSMIC (https://cancer.sanger.ac.uk/census)	Human genetic cancer association studies; Cancer attributes and hallmarks
Gene Expression Atlas (https://www.ebi.ac.uk/gxa)	Protein and mRNA expression in multiple species and tissues.
Genotype-Tissue Expression Portal (https://www.gtexportal.org)	mRNA expression in multiple species and tissues.
The Human Protein Atlas (https://www.proteinatlas.org/)	Protein and mRNA expression in normal and cancer tissues and cell lines; prognostic indicators for specific cancer types.
KEGG (https://www.genome.jp/kegg/)	Biological pathways
Reactome (https://reactome.org/)	Biological pathways
String DB (https://string-db.org/)	Biological pathways
